# The Evolving Role of Radiation Therapy in the Treatment of Biliary Tract Cancer

**DOI:** 10.3389/fonc.2020.604387

**Published:** 2020-12-14

**Authors:** Eleni Gkika, Maria A. Hawkins, Anca-Ligia Grosu, Thomas B. Brunner

**Affiliations:** ^1^ Department of Radiation Oncology, University Medical Centre Freiburg, Freiburg, Germany; ^2^ Medical Physics and Biomedical Engineering, University College London, London, United Kingdom; ^3^ Department of Radiation Oncology, University of Magdeburg, Magdeburg, Germany

**Keywords:** cholangiocarcinoma, biliary tract cancer, stereotactic body radiotherapy, chemoradiation, brachytherapy

## Abstract

Biliary tract cancers (BTC) are a disease entity comprising diverse epithelial tumors, which are categorized according to their anatomical location as intrahepatic (iCCA), perihilar (pCCA), distal (dCCA) cholangiocarcinomas, and gallbladder carcinomas (GBC), with distinct epidemiology, biology, and prognosis. Complete surgical resection is the mainstay in operable BTC as it is the only potentially curative treatment option. Nevertheless, even after curative (R0) resection, the 5-year survival rate ranges between 20 and 40% and the disease free survival rates (DFS) is approximately 48–65% after one year and 23–35% after three years without adjuvant treatment. Improvements in adjuvant chemotherapy have improved the DFS, but the role of adjuvant radiotherapy is unclear. On the other hand, more than 50% of the patients present with unresectable disease at the time of diagnosis, which limits the prognosis to a few months without treatment. Herein, we review the role of radiotherapy in the treatment of cholangiocarcinoma in the curative and palliative setting.

## Introduction

**Figure 1:**
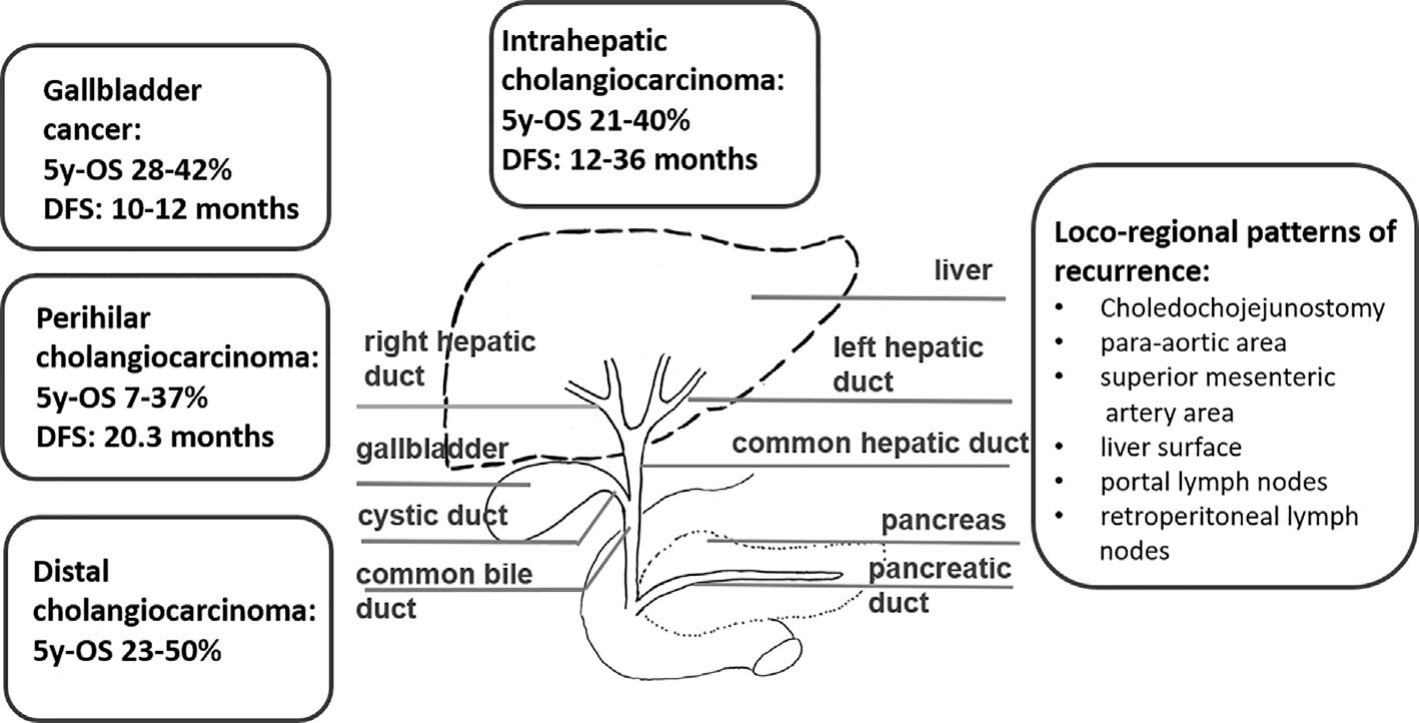
The classification of biliary tract cancers, overall survival and patterns of recurrence.

**Figure 2:**
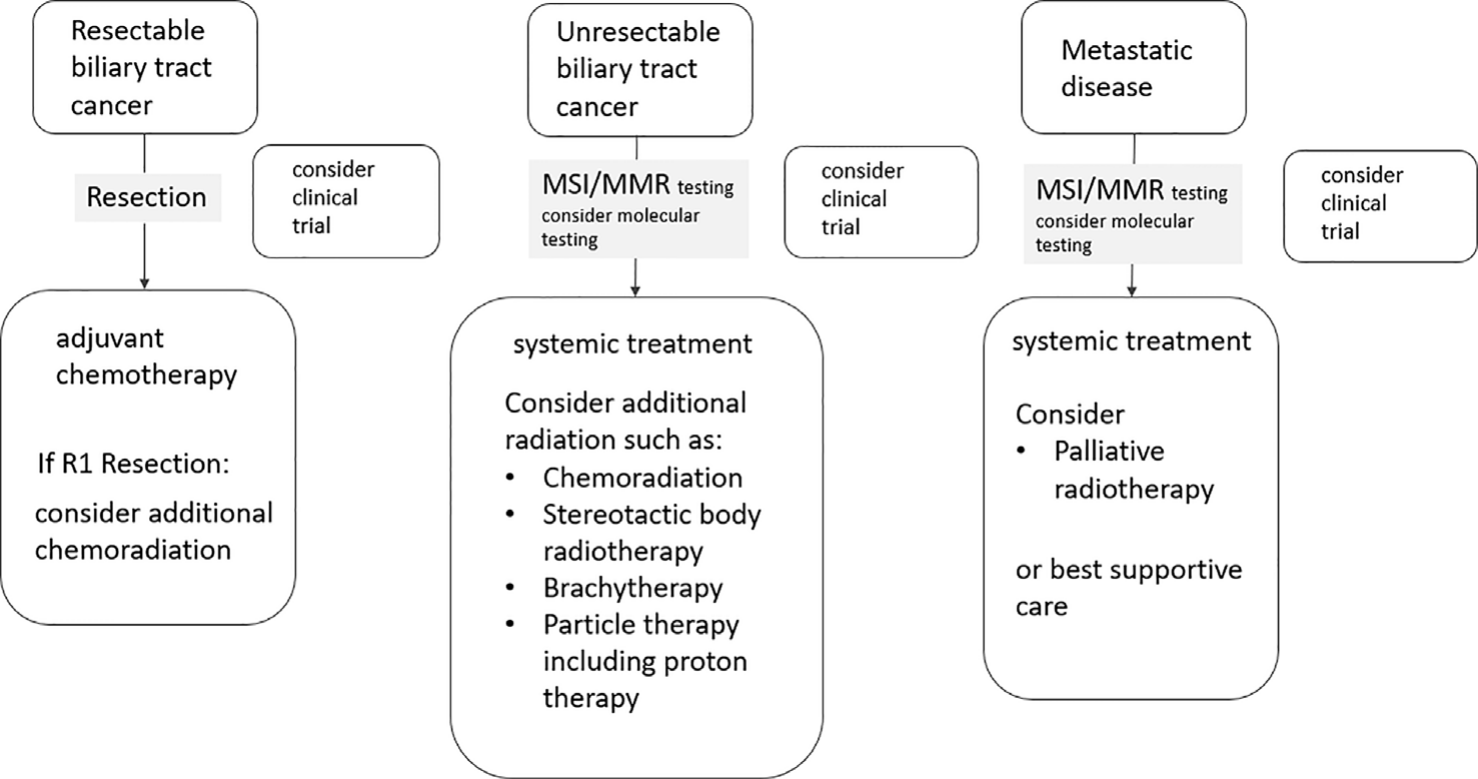
Flow chart illustrating the integration of radiotherapy in the treatment of biliary tract cancers.

Biliary tract cancers (BTCs) are the second most common hepatic malignancy after hepatocellular carcinomas comprising < 1% of all human cancers (1). They are sub-classified as intrahepatic cholangiocarcinomas (iCCA) originating from the biliary tree within the liver, and extrahepatic cholangiocarcinomas (eCCA) originating from the biliary tree outside the liver, and gallbladder carcinoma (GBC). eCCAs are further subdivided into perihilar (pCCA) and distal (dCCA) cholangiocarcinoma. Their geographical distribution is extremely variable, depending on their localisation, reflecting the difference in risk and genetic factors globally (2–8). BTCs are aggressive tumors and most patients are diagnosed in an advanced stage. More than 50% present with unresectable disease at the time of diagnosis, which limits the prognosis to a few months (9). For patients with operable BTC at diagnosis, complete surgical resection is the mainstay as it is the only potentially curative treatment option (1). Nevertheless, even after curative (R0) resection, the 5-year survival rates range between 20 and 40% (10–12, 13–15). The disease free survival rates (DFS) range between 48 and 65% after 1 year and 23 to 35% after 3 years, without adjuvant treatment (10–12, 13–15). Following surgical resection, both local recurrence and distant metastases occur frequently, with a relapse rate ranging between 56.5 and 88.4% in several prospective trials (16) (**Figure 1**). Several risk factors for disease recurrence after resection, such as positive margins, positive nodal status and/or vascular invasion, have been identified in several studies (11, 12, 17–21). For muscle invasive gallbladder carcinoma prognosis seems to be even worse than for cholangiocarcinoma (13). Due to the high rates of disease local recurrence and poor survival rates following radical surgery, postoperative treatment modalities, such as chemotherapy, radiotherapy, and chemoradiation, have been considered to improve survival after resection (22). On the other hand, patients with unresectable disease at the time of diagnosis are offered palliative chemotherapy according to guidelines, but radiotherapy could also play an important role. Herein, we review the role of radiotherapy in the treatment of cholangiocarcinoma in the curative and palliative setting (**Figure 2**).

## Methods

We conducted a systematic search of the PubMed library database published until June 2020 using the following search terms related to cholangiocarcinoma or gallbladder carcinoma and radiotherapy: (cholangiocarcinoma OR bile duct cancer OR Klatskin OR gallbladder) AND (radiotherapy OR chemoradiation OR radiochemotherapy OR chemoradiotherapy OR SBRT OR SABR OR stereotactic body radiotherapy OR stereotactic ablative body radiotherapy OR brachytherapy OR BT OR particle therapy OR proton therapy OR PBT). Additionally, we performed a search for ongoing unpublished trials in clinicaltrials.gov.

### Curative Treatment of BTC

In patients with iCCA the DFS ranges between 12 and 36 months in various studies with a 5 year overall survival (OS) between 21 and 40% and median OS as high as 80 months in one cohort after R0 resection (12, 23–26) (**Figure 1**). Routine lymphadenectomy at the level of the hepato-duodenal ligament is recommended during surgery according to international guidelines (1, 27). Some studies indicate that transplantation might be an effective option in patients with early iCCA. The size of the tumor, the grade, the presence of multiple lesions, vascular and/or perineural invasion, and the lymph node involvement were predictors of short DFS and should be reported by the pathologist to guide decisions regarding adjuvant therapy, although robust evidence for its use is lacking (1, 24).

In patients with pCCA the 5 year OS ranges between 7 and 37% in several studies (25, 26) (**Figure 1**). The resection often involves lobectomy bile-duct resection, right hemi-hepatectomy, regional lymphadenectomy, and Roux-en-Y hepaticojejunostomy (28). Several surgical advances have facilitated the resection of those tumors in the present years, while liver transplantation offers very good outcomes in selected patients with early disease (29). The presence of regional lymphadenopathy, although not an absolute contraindication for resection, is associated with inferior patient outcomes (28). Lymphadenectomy is at any case a standard part of every curative resection. Liver transplantation in unresectable cases has been explored in study following neoadjuvant chemoradiation, showing a 5-year DFS of 65% (30).

The 5-year OS rates for dCCA rage between 23 and 50% (25, 26) (**Figure 1**). Patients with dCCA typically undergo partial pancreaticoduodenectomy (Whipple procedure) with extended bile duct resection up to the hilum and dissection of the draining lymph nodes (1). In a large series, R0 resection was achieved in 78%.

GBC has two typical presentations: either (a) incidentally diagnosed in the histological workup of simple cholecystectomies or (b) as a symptomatic right upper quadrant tumor at an advanced stage (1). After R0 resection DFS ranges between 10 and 12 months and OS rates are about 55% after 1 year and about 30% after 3 years in patients with GBC following radical cholecystectomy and partial hepatectomy (10, 13–15) (**Figure 1**). Over the last 5 years, there is an improvement of the median 5 years OS rate from 28% (between 1995 and 2000) to 42% (between 2015 and 2020) (16). Patients with GBC tend to have higher rates of distant failure compared to CCA (31).

### Patterns of Recurrence and Prognostic Factors for Recurrence After Resection

Despite aggressive resection, at least 50% of patients experience recurrence of tumor with the mean time to recurrence ranging from 10 to 20 months (32), while in the major prospective studies evaluating chemotherapy vs. observation in the adjuvant setting, the incidence of relapse in the adjuvant chemotherapy arms ranged between 53.8 and 79.9% and in the observational arms between 56.5 and 88.4% (16). Some studies report a higher incidence of distant metastases in patients with GBC (31, 33), while in CCA relapse patterns vary significantly between studies. Recurrence most frequently involves intrahepatic metastasis, followed by simultaneous intra- and extrahepatic disease, and extrahepatic recurrence alone being the least common (31, 33, 34). Pathologic data suggest that high recurrence and low survival rates are, in part, a result of frequent and early portal vein, lymphatic, biliary and perineural invasion of tumor (35, 36) supporting a strong role for aggressive multimodal therapy. In a study by Jung et al. (37) relapses were the most frequent in the choledochojejunostomy (17.7%); para-aortic area(16.1%) and superior mesenteric artery area (16.1%); and portal vein area (14.5%). In a further study (38) patients who did not receive adjuvant RT developed loco-regional recurrences in 51%, primarily at biliary anastomosis/liver surface, portal lymph nodes, and retroperitoneal lymph nodes. Concerning the patterns of recurrence after curative resection for GBC many patients developed distant recurrences, although the most common site of recurrence was the liver (n = 22, 34.4%) followed by the peritoneum (n = 10, 15.6%) (39). In a study of 156 patients (80 with GBC and 76 with HCCA) Jarnagin et al. (13) reported that 52 (68%) patients with hCCA and 53 (66%) patients with GBC had disease recurrence at a median follow-up of 24 months. The median time to disease recurrence was shorter for patients with GBC compared with patients with hCCA (11.5 vs. 20.3 months; P = 0.007). Of those who developed disease recurrence, isolated loco-regional disease as the first site of failure occurred in 15% of patients with GBCA compared with 59% of patients with hCCA (P < 0.001).

Factors associated with increased risk of relapse include the presence of R1, high serum carbohydrate antigen (CA) 19–9 and the presence of lymph node metastases (16, 23, 40, 41). In other studies additional factors have been identified such as a tumor size >5 cm, the number of lesions, vascular invasion, tumor grading, obstructive jaundice, a neutrophil to lymphocyte ratio (NLR) < 5, and lack of perineural invasion (12, 17–20, 25, 32, 34, 42–46). In a meta-anlysis by Ke et al. (47) the hazard ratio (HR) was 0.60 (95% CI = 0.51–0.69) in the positive resection margin group, and 0.67 (95% CI = 0.57–0.76) in lymph node metastasis (LNM) group. The effect of adjuvant treatment (AT) on the patients with LNM was evaluated in 4 included cohorts (48–51). Using a random-effect model, the pooled HR for the OS in the AT group was 0.67 (95% CI 0.57–0.76), compared with the non-AT group.

Concerning the resection margins, DeOliveira et al. (24) studied 564 consecutive patients treated between 1973 and 2004 (42% distal, 50% perihilar 8% intrahepatic). Whilst the negative margin rate increased during the period studied, the survival of patients with positive margins was worse and on multivariate analysis patients with R0 and N0 had a statistically significant better survival. Additionally Farges et al. reported that surgical margins less that 1mm had a similar outcome compared to R1 resections and margins greater than 5mm were associated with improved survival (52). In a study by Tamandl et al. (11) the distance between the tumor and resection margins correlated with the median DFS ranging between 11.4 to 9.8 months, while in case of R1 resection, the median disease free survival was 9.9 months. A retrospective study evaluating the results of surgical therapy for intrahepatic CCA showed that the most frequent site of disease recurrence was the liver (53).

For patients diagnosed with eCCA, the presence of postoperative CA19-9 (HR 2.26) and presence of lymph node infiltration (HR 2.33) were associated with worse outcomes. Patients with resected eCCA with high pre-and post-operative CA19-9 were shown to have a higher distant metastasis rate and shorter disease-free interval (40). Involvement of adjacent structures, perineural invasion, and poorly-differentiated histology has also been associated with poor outcomes for resected eCCA (16, 23, 41, 54–56). Five-year survival of N+ versus N0 disease was 0 to 29% versus 32 to 67% in pCCA, and 16 to 21% versus 42 to 61% in dCCA (26).

For iCCA, factors associated with increased relapse rate and poor prognosis include R1 resection, lymphatic invasion, vascular invasion, and peri-ductal infiltrating disease (23, 42, 57–60). Prognostic nomograms have been designed for patients with resected iCCA (61) including serum carcinoembryonic antigen (CEA), CA199), tumor diameter and number, vascular invasion, lymph node metastasis, direct invasion, and local extra-hepatic metastasis, showing a superiority in prognostic discrimination compared to five other staging systems for iCCA (p < 0.001). Five-year survival of N+ versus N0 disease was 0 to 9% versus 36 to 43% in iCCA (26).

For GBM, higher recurrence rates are associated with R1-resection, depth of mural invasion, lymph node metastasis, extramural extension, and perineural invasion (16, 1, 62).

### Adjuvant Therapies

The high rates of recurrence following surgery justify the consideration of an adjuvant treatment. In a meta-analysis by Horgan et al. (63), including 6712 patients treated between 1960 and 2010 there was a trend for improved OS with adjuvant treatment compared to resection alone (odds ratio (OR), 0.74; P = 0.06). Chemotherapy regimens, either alone or in combination with radiotherapy, showed a statistically greater impact on survival than radiotherapy alone, especially for patients with lymph node involvement (OR, 0.49; P = 0.004) and involved resection margins (OR, 0.36; P = 0.002). Manterola et al (64). conducted a meta-analysis including 3 systematic reviews and 24 observational studies evaluating the role of adjuvant treatment in GBC concluding that the results do not provide strong evidence that AT is effective in patients who undergo resection for GBC. Subgroups with positive lymph nodes and positive surgical margins may have a survival advantage. Additionally, in the meta-analysis by Ke et al. (47), subgroup analysis showed that the pooled HR for the OS rate in the AT group compared with non-AT group were as follows: chemotherapy group was 0.57 (95% CI = 0.44–0.70), TACE group was 0.56 (95% CI 0.31–0.82), radiotherapy group was 0.71 (95% CI = 0.39–1.03), chemoradiation group was 0.73 (95% CI = 0.57–0.89), positive resection margin group was 0.60 (95% CI = 0.51–0.69), and lymph node metastasis (LNM) group was 0.67 (95% CI = 0.57–0.76). Thus, prospective trials are needed to elaborate the role of adjuvant therapy.

### Adjuvant Chemotherapy

Over the last decades, four randomized phase III clinical trials have evaluated the role of adjuvant chemotherapy in resected BTC (**Table 1**). In the BCAT study patients with eCCA were randomized between observation alone vs. gemcitabine. Patients with CCA and GBC were randomized between observation vs. gemcitabine and oxaliplatin in the PRODIGE-12/ACCORD-18 trial, observation vs. capecitabine in BILCAP study and observation vs. mitomycin C combined with 5 FU in the study by Takada et al. (10).

**Table 1:** Phase III trials exploring the role of adjuvant Chemotherapy.

	Takeda (2)	BCAT (65)	Prodige-12/ACCORD-18 (66)	BILCAP (67)
Study arms	Mitomycin and 5 FU (MF) vs. S alone	Gemcitabine (Gem) vs. S alone	Gemcitabine and Oxaliplatin (Gemox)vs. S alone	Capecitabine (Cap) vs. S alone
Sample size	508	226	196	447
BTC subtype	GBC:139CCA:140	hCCA:102dCCA:123	CCA/GBC	CCCA/GBC
Incidence of recurrence	GCB: 80% (S+MF) 88% (S)	54% (S+Gem) vs. 57% (S)	62% (S+GemOX) vs. 68% (S)	60% (S+Cap)
Localisation of recurrence	n.r.	Liver, local, peritoneal, lymph nodes		n.r.

S, surgery; n.r., not reported.

In the prospective randomized trial by Takada there was a non-significant benefit for patients with CCA with R0 resection receiving adjuvant therapy, with a DFS at 5 years of 15.8 vs. 32.4% and an OS at 5 years of 28.3 vs. 41.0% (10). The PRODIGE-12/ACCORD-18 trial showed a non-significant improvement in the recurrence-free survival (RFS) for gemcitabine and oxaliplatin compared to observation alone (30.4 vs. 22 months, HR 0.83, 95% CI: 0.58–1.19, p = 0.31) in 196 patients (66), while the BCAT trial has shown that adjuvant gemcitabine is not associated with improved RFS or OS (65). the BILCAP study (68)showed a benefit from adjuvant capecitabine in terms of OS (pre-planned sensitivity analysis in the intention-to-treat population and in the per-protocol analysis), with confirmed benefit in terms of RFS. The treatment was well tolerated without unexpected adverse events or a detriment in quality of life. Based on the BILCAP trial, international guidelines recommend adjuvant capecitabine for a period of 6 months following potentially curative resection of CCA as the current standard of care for resected CCA and GBC (16).

### Adjuvant Radiotherapy

#### Chemoradiation

Several studies have evaluated the role of adjuvant radiotherapy or chemoradiation (CRT) in this setting. A systematic review and meta-analysis on adjuvant radiotherapy in EHCC (69) including 10 studies, demonstrated an improvement in OS with adjuvant radiation therapy or chemoradiation with 5 FU (57% of the studies) (HR, hazards ratio 0.62, 95% CI: 0.48–0.78, *p* < 0.001) with a low incidence of late radiation toxicity (2–9% of the patients), mainly late obstruction or gastrointestinal bleeding. In a further systematic review and meta-analysis including 20 studies (63, 70), patients receiving CT or CRT derived statistically greater benefit than RT alone (OR, 0.39, 0.61, and 0.98, respectively; P = 0.02), especially patients with LN-positive disease (OR, 0.49; P = 0.004) and R1 disease (OR, 0.36; P = 0.002). While in a further meta-analysis by Ren et al. (71) including 21 studies, with 1465 EHCC and GBC patients, 5-year overall survival (OS) rate was higher in the adjuvant RT group than in the non-RT group (OR = 0.63; 95% CI = 0.50–0.81, p = 0.0002). The 5-year OS rate was significantly higher for those with positive lymph nodes (OR = 0.15; 95% CI = 0.07–0.35; p < 0.00001) and margin-positive resection (OR = 0.40; 95% CI = 0.19–0.85; p = 0.02) in the adjuvant RT group than in the non-RT group. The local recurrence rate was significantly lower in the adjuvant RT group than in the non-RT group (OR = 0.54; 95% CI = 0.38–0.76, p = 0.0004).

Four national cancer database (NCD) analyses and four Surveillance, Epidemiology and End Results (SEER) database analyses have evaluated the role of adjuvant treatment, including radiation. In an analysis from 1998–2013 (72), 2897 patients were identified, R0 status was achieved in 1951 patients (67.3%) and RT was delivered to 525 patients (R0 = 255, R1/R2 = 230, unknown = 43). Following propensity score matching, the OS for R0 versus R1/R2 resection was 31.2 versus 19.5 months (p = .001), respectively. RT was associated with a trend toward improved survival for R1/R2 lymph node negative patients (39.5 vs. 21.1 months; p = 0.052). Patients with a positive resection margin had a higher risk of disease recurrence (HR, 1.61; 95% CI, 1.15–2.27; p = .01) and a shorter overall survival (HR 1.54; 95% CI, 1.12–2.11; p = 0,001). In an additional NCD analysis, from 2004–2012 (73), evaluating the role of surgery and adjuvant therapy in lymph node positive GBC and iCCA, adjuvant treatment, including radiation, was associated with a lower risk of death relative to surgery alone for patients with GBC regardless of margin status (margin-negative resection: HR, 0.66; 95% CI, 0.52–0.84; margin-positive resection: HR, 0.54; 95% CI, 0.39–0.75), while adjuvant chemotherapy alone was not. For patients with iCCA, no survival benefit was detected with adjuvant chemotherapy or radiation for those who underwent either margin-positive or margin-negative resection. In a further NCDB analysis (74), evaluating the benefit of adjuvant therapy following resection for intrahepatic cholangiocarcinoma after adjusting for other prognostic variables, patients were found to significantly benefit from AT if they had positive lymph nodes (chemotherapy: HR, 0.54; p = 0.0365; chemoradiation: HR, 0.50, p = 0.005) or positive margins (chemotherapy: HR, 0.44; p = 0.0016; chemoradiation: HR, 0.57; p = 0.0039). Lastly, in a propensity score matched analysis from a NCD (2004–2014) including extrahepatic bile duct cancers adjuvant therapy was associated with improved median OS for hilar tumors (40.0 vs. 30.6 months; p = 0.025) but not distal tumors (33.0 vs. 30.3 months; p = 0.123), while chemoradiation was associated with superior outcomes compared with chemotherapy alone in the subset of margin-positive resection (HR 0.63; 95% CI, 0.42–0.94) (75).

A SEER database comprising patients with EHCC (*n* = 1569) treated between 1973 and 2005 suggest an early survival advantage for adjuvant radiotherapy (25 vs. 21 months after R1 resection with versus without adjuvant radiotherapy, p < 0.001) whereas survival was almost identical for patients after R0-resection (26 vs. 25 months) (76). In another SEER analysis by Shinohara including 4,758 patients palliative RT prolonged survival, while the benefit associated with surgery and RT was significant on univariate analysis but not after controlling for potential confounders using the propensity score (77). A further SEER database analysis (78) including 3839 patients with IHCC, use of surgery, and adjuvant radiation therapy conferred the greatest benefit on OS (HR = 0.40; 95% CI, 0.34– 0.47), followed by surgery alone (hazard ratio [HR], 0.49; 95% CI, 0.44–0.54) and radiation therapy alone (HR, 0.68; 95% CI, 0.59–0.77) compared with no treatment on multivariate analysis. Finally, in a SEER analysis from 1973–2003, including patients with resected eCCA, adjuvant RT was not associated with an improvement in long-term overall survival in patients with resected extrahepatic bile duct cancer (79). Major limitations of these four SEER database analyses are the lack of information concerning patient and treatment related factors and subsequent treatments. Furthermore, most of these patients were treated without concurrent chemoradiation.

In several studies maintenance chemotherapy after adjuvant concurrent CRT showed promising results (80–82). In a Phase II study in pancreatic cancers and BTCs (83), evaluating the combination of adjuvant chemotherapy with taxane and gemcitabine followed by chemoradiation the treatment was discontinued by 15% of the patients due to adverse events. Grade 3 or greater non-hematological toxicities were observed in 15% of patients. Recently the Phase II SWOG 0809 trial (84) evaluated the role of adjuvant gemcitabine with capecitabine followed by concurrent CRT with capecitabine in patients with BTC mainly EHCC and GBC. The OS was 35 months, similar for both R0 and R1 resected patients (R0, 34 months; R1, 35 months). The trial met the primary endpoint, the treatment incurred toxicity grade 3 to 4 adverse effects such as neutropenia (44%), hand-foot syndrome (11%), diarrhoea (8%), lymphopenia (8%), and leukopenia (6%). This trial establishes the feasibility of conducting national adjuvant trials in EHCC and GBCA and provides baseline data for planning future phase III trials (85).

In conclusion, non-randomized phase II trial and meta-analyses support the efficacy chemoradiation in the adjuvant setting. Selected prospective and retrospective studies with subgroups of patients receiving adjuvant radiotherapy or chemoradiation are summarized in **Table 2**.

**Table 2:** Selected results after adjuvant radio (chemo)therapy in cholangiocarcinoma.

	Design	Nr of Patients Total/RT)	RTx	CTx	R0	OS(months)
Gonzales (86)	R	55/23	Aprox. 42.7–55.6 Gy ± BT	5 FU (11)	n.r.	S: 8.25 S+RT: 19
Schoenthaler (87)	R	129/67	RT 54 Gy or CP	5 FU	n.r.	S:11 † S+RT:21.5† S+CP:61†
Pitt (88)	P	50/23	46 (40–60) Gy + 13 (2–18)Gy BT	none	R0:42%	S: 20 S+RT: 20
Zlotecki (89)	R	46/30	30–60 Gy ± BT	none	n.r.	S:26.1 S+RT:43.4
Todoroki (90)	R	63/28	43.6 Gy 21 Gy IORT	n.r.	25%	S:10 † S+RT:32†
Serafini (91)	R	92/34	39.6–50.4 Gy	yes	n.r.	S: 29 S+RT:42
Kim (92)	R	84/84	40–45 Gy	5 FU	R0:47 R1:25	R0:36%@5years R1:35%@5years R2:0
Nakeeb (93)	R	140/55	n.r.	5 FU or gemcitabine	n.r.	S:27.8
Gerhards (94)	R	91/71	46 Gy ± BT	none	n.r.	S: 8 S+RT:24
Heron (95)	R	118/23	46 (27–60)Gy + 25 (9–33) Gy BT	5 FU	n.r.	Proximal S+RT:24 Distal S:62.5
Lindel (96)	R	20/10	EBRT ± IORT	None	n.r.	S:20.2 S+RT:28.8
Itoh (97)	R	37/16	52.3 Gy	n.r.	R0:8 R1:9 R2:4	eCCA: S:16 S+R:17 GBC: S:34 S+RT:13
Czito (98)	R	22/22	45 Gy	± 5 FU	n.r	S+r:22.8
Sagawa (99)	R	9/39	20–50 Gy ± BT	none	R0:34 R1/2:35	S:33.3%@3years S+RT:40.9%%@3years
Schoppmeyer (81)	Ph I/II	18	49.6 Gy	± gemcitabine	R0:5 R1:1 R0:12	R2:7.9months R0/1:not reached All:11months
Ben-David (100)	R	81/81	58.4 Gy	± 5FU	R0:12 R1:16 R2:51	R0:24.1 R1:n.r. R2:13.1
Mahantshetty (101)	R	60/40	50 Gy	± 5 FU, mitomycin	R0:90%	All: 25%@5 years
Dinant (102)	R	99/99	55 Gy	none	R0/1:83 R2:16	S+R:27%@5years
Cheng (103)	R	75/23	50Gy	n.r.	n.r.	All:35.5
Oh (38)	R	60/60	45–55Gy	5FU or gemcitabine	R0:24 R1:23 R2:13	R0:53%@2years R1:40.7%@2years
Hughes (80)	R	34/34	50.4–54Gy	± 5 FU	n.r.	S+R: 36.9 35%@5years
Borghero (104)	R	65/42	55 Gy ± BT ± IORT	± 5 FU or capecitabine	n.r.	S:31 S+RT:32
Shinohara (77)	R	4,758 701	n.r.	n.r.	n.r.	S:4 S+R:16
Shinohara (78)	R	3839 /286	n.r.	n.r.	n.r	S:3 S+R:11
Gold (105)	R	73/25	50.4	5 FU	n.r.	S:50.4 S+R:57.6
Lim (82)	R	120/120	40–54 Gy	5 FU	R0:66% R1:34%	S+R:30.8%@3years S+R:62.6%@3years*
Nelson (106)	R	45/33	50.4 Gy ± BT boost	Mostly 5 FU	R0:36 R1:6 R2:3	S+R:34 months
Jiang (48)	R	90/24	50 Gy	none	n.r.	S:9.5 S+RT:19.1
Vern-Gross (79)	R	1491/473	n.r.	n.r.	n.r	All: 20 months
Beltran (107)	R	23/23	45–60 Gy	5 FU	R0:13 R1:8 n.r:2	36%@5 years (R0:61%@5years)
Kim (108)	R	168/115	45 (45–55.8) Gy	yes		S:28.2%@5 years S+R:36.5%@5 years
Cho (83)	Ph II	48/30	45Gy	5 FU	n.r.	23¥
Ben-Josef (84)	Ph II	79/79	52.5–59.4	capecitabine	R0:54 R1:25	R0:34 R1:35
Hayashi (109)	R	187/187	50.4 Gy	Gemcitabine or 5 FU	R0:21% R1:48% R2:20%	S+R: 15
Sur (74)	R	638/147	n.r.	n.r.	R0:504 R1:179	n.r.
Hammad (72)	R	2897/525	n.r.	yes	R0:255 R1/2 = 230	S:21.1† S+RT:39.5†
Dover (110)	R	95/23	50.4–54 Gy	5 FU	R0:67 R1:	S:26.3 S+RT:30.2
Im (111)	R	336/78	50.4 Gy	± 5 FU or gemcitabine	R0:251 R1:67 R2:18	S:43.2 S+RT:42.9 S+CRT: 47.6
Kim (112)	R	132/132	50.4 Gy	± 5 FU or capecitabine	R0:118 R1:14	S+R:48.1
Ecker (75)**	R	1718/859	n.r.	n.r		S:29.6 S+CT ± RT:36
Tran (73)	R	1210/358	n.r.	n.r.	n.r	GBC S:13.3 S+RT:24.7
Gu (113)**	R	78/39	n.r.	yes	n.r.	S:13 S+RT:27
Lee (114)	R	84/32	47.4–54 Gy	± 5 FU ± cisplatin	R0:52 R1:32	R0:S:61.5 R1:S+CRT:57.9 R1:S+RT:15.4
Zheng (115)	R	70/26	50–60 Gy	none	R0:21 R1:49	R0:S:65%@3 years R1:S:20%@3 years R1:S+RT: 55%@3 years
Mukai (116)	R	32/32	50Gy	gemcitabine	R0:0 R1:16 R2:16	S+R:40

Fx, Fraction; 5-FU, 5-fluorouracil; n.r., not reported; BT, Brachytherapy; IORT, intraoperative brachytherapy; CP, charged particles; S, surgery; S+ RT, radiation therapy; CTx, chemotherapy; RTx, radiotherapy; CT, chemotherapy.

*in patients with concurrent chemoradiation followed by chemotherapy.

**Propensity score matching.

^†^for R1 resected.

¥biliary cancer.

#### Brachytherapy

The role of brachytherapy (BT) mostly as brachytherapy boost after EBRT has been also evaluated in the adjuvant setting mostly in singe-centre retrospective studies. Gerhards (94) et al. reported that the addition of BT to external radiotherapy in the adjuvant setting provided no significant benefit in hCCA, while the incidence of toxicities was higher. In the case of R1 resection, a combination of adjuvant therapy with EBRT plus BT led toa comparable survival as in patients with R0 resection in hCCA with a median survival of 26 months in a small number of selected patients (117). In **Table 2**, we summarize the results of adjuvant RT with or without brachytherapy boost. In conclusion, the additional advantage through a BT boost in the adjuvant setting is unclear.

#### Neoadjuvant Chemoradiation

Neoadjuvant chemoradiation has been investigated either before resection or prior to liver transplantation (118–120) as a treatment option in primarily unresectable cholangiocarcinoma.

Chemoradiation prior to surgery was evaluated in four studies (**Table 3**), leading to R0 resection rates between 71.4 and 100%. Nelson et al. (106) compared retrospectively patients treated with adjuvant vs. neoadjuvant chemoradiation showing that neoadjuvant chemoradiation led to a prolonged OS (5-year survival 53 vs. 23%, *p* = 0.16) and similar rates of Grade 2–3 surgical morbidity (16 vs. 33%, *p* = 0.24) compared with those treated in the postoperative setting, although the latter presented with more advanced disease at diagnosis. A Phase I trial estimated the maximum tolerated dose of gemcitabine at 600 mg m^−2^ with 45 Gy in 1.8-Gy daily fraction for neoadjuvant CRT (124).

**Table 3:** Neoadjuvant chemoradiation of advanced cholangiocarcinoma.

Authors	Study	Nr of Patients	EBRT	BT	Chemotherapy	R0 Resection	Median OS
McMasters (121)	P	hCCA:5 dCCA:4	45–50.4 Gy	none	5-FU	100%	n.r.
Nelson (106)	R	h+dCCA:12	50.4 Gy	done	5-FU	91%	34 months
Jung (122)	R	hCCA:12	45–50.4 Gy	none	5-FU/ Gemcitabine	83,3%	32.9 months
Sumiyoshi (123)	R	hCCA:15	50 Gy	none	S1	71.4%*	37 months*

EBRT, external beam radiotherapy; BT, Brachytherapy; bid, twice daily; LT, liver transplantation; SBRT, stereotactic body radiotherapy; 5-FU, 5-fluorouracil; OS, overall survival

*for the 11 patients who underwent surgery.

Neoadjuvant chemoradiation was also used in combination with liver transplantation in patients with pCCA in small single centre studies, either as brachytherapy (125), SBRT (126), or concurrent CRT (127–130) (**Table 4**). In a multi-centre study by Darwish Murad et al. (30) with pCCA treated with neoadjuvant therapy followed up by liver transplantation at 12 US centers, the recurrence-free survival after 5 year was 65% showing this therapy is effective in selected patients. Both concepts should be further evaluated in clinical trials in addition to ongoing trial of Liver Resection versus Radio-chemotherapy-Transplantation for Hilar Cholangiocarcinoma (TRANSPHIL NCT02232932).

**Table 4:** Neoadjuvant radiotherapy followed by liver transplantation.

Authors	Patients	EBRT	BT	Chemotherapy	OS
De Vreede (127)	19	45 Gy/1.5Gy/bid	± 20–30 Gy 192Ir	± 5-FU	n.r.
Heimbach (128)	46	45 Gy/1.5Gy/bid	± 20–30 Gy 192Ir	± 5-FU	82% @5 years after LT
Sudan (125)	11	none	± 60Gy 192Ir	± 5-FU	45% alive @7.5 years
Wu (131)	6	44 Gy in 22 frs	± 30 Gy 192Ir	± yes	n.r.
Murad (30)	287	Dose n.r.	± BT	± yes	53% @5 years after LT
Welling (126)	17	SBRT 50–60 Gy/3–5 fractions	none	capecitabine	83% @1 year after LT
Mukewar (130)	40	45 Gy/1.5 Gy/bid	± 9.3–16 Gy in 1–4 Fx	5-FU or capecitabine	n.r.

EBRT, external beam radiotherapy; BT, Brachytherapy; bid, twice daily; LT, liver transplantation; SBRT, stereotactic body radiotherapy; 5-FU, 5-fluorouracil; OS, overall survival; Fx, fractions.

n.r., not reported; OS, overall survival.

### Management of Locally Advanced Disease

#### Systemic Treatment

Systemic treatment is the treatment of choice for unresectable BTC according to guidelines (1). Earlier randomized, controlled studies have shown that chemotherapy improves survival in patients with advanced BTC compared with best supportive care (132–134). In a pooled analysis of 104 studies in advanced BTC, gemcitabine combined with cisplatin or oxaliplatin resulted to the best response rates, however, without significantly improving survival (135). In the phase III UK ABC 02 study Valle et al. (9) reported a median survival close to a year (11.7 months) for cisplatin/gemcitabine, compared with 8.1 months for gemcitabine alone(95% CI: 0.53–0.79; P < 0.001) these results were also confirmed in the BT22 study (136) and in a subsequent meta-analysis (137). Therefore, the combination of cisplatin and gemcitabine is currently regarded as standard of care in metastatic or unresectable BTC. Other treatments tested in randomized trials include the combination of gemcitabine, cisplatin and nab-paclitaxel (138), modified folfirinox vs. cisplatin gemcitabine (PRODIGE38-AMEBICA trial) (139), or nal-IRI, 5 FU, leucovorin vs. cisplatin gemcitabine (NIFE trial) (140).

Concerning the role of targeted therapies, although initial results from a single-arm study using cetuximab in combination with GemOX were promising (141), there was no benefit observed in a subsequent randomized phase II study (142). Similar negative findings were observed with erlotinib or panitumumab, sorafenib, or cedira-nib (an oral VEGFR-1, −2, and−3, PDGF, and c-Kit tyrosine kinase inhibitor) and the cisplatin/gemcitabine combination (1, 143, 144). In a phase II trial, regorafenib showed a disease control rate of 56%, indicating that it might be useful in refractory disease (145). Moreover, recently described gene fusions and mutations are being investigated. Emerging therapies that hold considerable promise include FGFR inhibitors such as pemigatinib and IDH1 and/or IDH2 inhibitors (29, 146, 147), whereas the inhibition of other molecular pathways, including the RAS/RAF/MEK/ERK, the MET, the PI3K/AKT/mTOR and angiogenetic pathways, is unclear (148). Certain tumor genetic aberrations have been associated with a likelihood of response to immune-checkpoint inhibitors, which might relate to the expression of neoantigens capable of eliciting an antitumor T-cell response (29). Several checkpoint inhibitors are currently being evaluated in a large number of clinical trials either as monotherapy or dual checkpoint inhibition but also in combination with chemotherapy or molecular targeted therapies (29). In some studies, it was indicated that tumors with DNA mismatch repair deficiency (dMMR) are sensitive to PD-1 blockade, so that for tumors with microsatellite instability (MSI-high) or dMMR tumors progressing after prior treatment, pembrolizumab is a possible treatment option (149–151).

#### Definitive Chemoradiation

For locally advanced inoperable cholangiocarcinoma definitive chemoradiation in has been evaluated in several prospective and retrospective studies (**Table 5**). Radiotherapy improved cancer-specific survival in inoperable patients (P <.0001) in a SEER database analysis (174). The French FFCD trial (167) randomized patients with hilar or extrahepatic non-metastatic BTC between chemoradiation (50 Gy with concurrent cisplatin and 5-FU) or chemotherapy with gemcitabine and oxaliplatin (GemOx). The trial was closed before completion due to slow recruitment after 34 patients had been enrolled, showing that GemOx was at least as efficient as chemoradiation. Most studies were conducted in combination with 5 FU, gemcitabine, or cisplatin with a median radiotherapy dose of ca 50 Gy, leading to an actuarial 2-year LC of 29.0% in one study and a PFS between 6.8 and 10.5 months (median: 7.5 months) (163, 164, 166, 167, 169–171), while in other studies, dose escalation led to higher LC rates and improvement of OS (175). Tao et al. (175) reported a median survival of 30 months for all patients (1-, 2-, and 3-year OS was 87, 61, and 44%, respectively). Patients with a higher biological effective dose (BED) of 80.5 Gy had an improved local control (LC: 78 vs. 45% after 3 years, p = 0.03) and overall survival (median OS: not reached vs. 27 months p = 0.02) compared to patients with lower doses. Patients receiving a BT boost had a better LC compared to patients with EBRT without BT (97 vs. 56% at 1 year) (165). OS ranged between 9.6 and 13.5 months (median: 13 months, **Table 5**), with acceptable toxicity mostly grade ≥ 3 acute hematological and/or gastrointestinal toxicity (163, 164, 166, 167, 169–171), while in some cases, the use of a BT boost resulted to better LC rates (2 year LC 53 vs. 25%) (170).

**Table 5:** Radiotherapy in inoperable cholangiocarcinoma.

Author	Design	Nr of patients	Radiotherapy	Chemotherapy	OS (median)	LC
Fields (152)	R	17	50.4 (44–63) Gy ± BT	n.r.	10.5 months	n.r.
Veeze-Kuijpers (153)	R	31	40 Gy ± BT Boost 25 Gy	n.r.	10 months	n.r.
Buskirk (154)	R	34	50–60 Gy ± BT ± IORT	5 FU	12 months	n.r.
Alden (155)	R	40	46 Gy ± BT 25 Gy	5 FU Mitomicyn C	9 months	n.r.
Fritz (156)	R	30	30–45 Gy +20–45 Gy BT	none	10	n.r.
Foo (157)	R	24	50.4 Gy ± BT Boost 20 Gy	5 FU	12.8 months	n.r.
Morganti (158)	R	20	39.6–50.4 Gy + 30–50 Gy BT	5 FU	21 months	33 months*
Crane (159)	R	52	30–85 Gy ± BT Boost 20 Gy or IORT 20 Gy	5 FU	10 months	10 months
Shin (160)	R	31	50.4 Gy + 15 Gy BT	none	22% @ 2years	71% @ 2 years
Takamura (161)	R	93	50 Gy ± BT Boost 39.2 Gy	n.r.	12 months	n.r.
Brunner (162)	R	98	50.8 Gy	5 FU, gemcitabine	11.8 months	n.r.
Ben-David (100)	R	52	58.4 (23–88.2)Gy	5 FU, gemcitabine	13.1	n.r.
Shinohara (78)	R	475	n.r.	n.r.	9 months	n.r.
Baisden (163)	Ph II	10	50 Gy	capecitabine	13 months	n.r.
Fuller (76)	R	146	n.r.	n.r.	12.18	n.r.
Moureau-Zabotto (164)	R	30	48.25 Gy (30–78 Gy)	5 FU, cisplatin, capecitabine	12 months	n.r.
Ghaafori (165)	R	37	45 Gy + 25 Gy BT	none	59%@1 year 22%@2 years	90%@ 1 year
Yi (166)	R	106	50.4 Gy	5 FU, gemcitabine	10.7 months	n.r.
Phelip (167)	Ph II	18	50 Gy	5 FU, cisplatin	13.5 months	n.r.
Tao (168)	R	79	58.05 (35–100) Gy	n.r.	30 months	81%@1year 23 months
Chen (169)	R	34	55.1 Gy	± 5 FU	13.5 months (cCRT) 6.7 (RT)	n.r.
Autorino (170)	Ph II	27	50 Gy +15 Gy BT	gemcitabine	27%	29%
Lee (171)	Ph II	18	45 Gy	gemcitabine	9.6 months	n.r.
Elganaimy (172)	R	80	30–75 Gy	Capecitabine or 5 FU	18.7	77.4 median
Aghili (173)	R	38	45–50.4 Gy + 20 Gy BT	capecitabine	15	n.r.

R, retrospective; Ph, Phase; BT, Brachytherapy; cCRT, concurrent chemoradiation; RT, radiotherapy.

*time to local progression.

In conclusion, BTCs might need higher doses in order to achieve a better local control and maybe also a survival benefit. Concepts for safer dose escalation include the use of stereotactic body radiotherapy (SBRT), brachytherapy (BT), or proton beam radiation therapy (PBRT).

#### Stereotactic Body Radiotherapy

There is emerging evidence concerning the efficacy of stereotactic body radiotherapy (SBRT) in the treatment of inoperable BTC. Several prospective and retrospective studies SBRT led to local control rates ranging between 65 and 100% with a median OS of 11–35.5 years (median 15 months), in selected patients (176–178). In a systematic review, including 10 studies and 231 patients, the pooled 1 year LC was 83.4% (95% CI: 76.5–89.4%) (178). According to the anatomical location of CCA, 1 year OS was 57.1% (range: 45.0–58.0%), 81.5% (range: 80.0–83.0%), and 58.7% (range: 45.0–73.0%) in studies including iCCA, eCCA, and both sites, respectively (126, 178–187).

Furthermore, in several studies dose escalation correlated with prolonged OS and LC (175, 177). In a study by Brunner et al. LC rates at 12 and 24 months were 91% and 80% for BED_max_ >91 Gy_10_ vs. 66 and 39% for lower doses (*p* = 0.009) (177). Additionally SBRT is a well-tolerated treatment with a low incidence of toxicities <10% (178, 188), while in a meta-analysis (178), only one case of fatal liver failure was reported in one patient despite compliance with dose/volume constraints. Additionally, SBRT has the advantage of being easily incorporated in systemic treatments showing high rates of OS after 1 year (median: 73.0%; range: 58.0–80.0%) (181, 185, 189) or even as neoadjuvant treatment in combination with capecitabine followed by liver transplantation leading to a 1 year OS of 83% as previously reported (126) (**Table 6**). The addition of stereotactic body radiotherapy to systemic chemotherapy in locally advanced biliary tract cancers is being investigated in a randomized phase II trial (ABC07(ISRCTN10639376) (https://doi.org/10.1186/ISRCTN10639376).

**Table 6:** SBRT in the treatment of cholangiocarcinoma.

Authors	Study	Nr of Lesions	Nr. of Fractions	Total Dose (Gy)	LC@ 1year	Median OS (months)	Late Toxicity
Herfarth (190)	P	CCA:3	1	14–26	81%@18 months†	n.r.	No grade 3
Tse (191)	P	iCCA:10 eCCA:0	6	28–48	65%	15	1 biliary obstruction 1 bowel obstruction
Goodman (192)	P	iCCA:5 eCCA:0	1	18–30	77%	28.6	None
Goyal (193)	R	iCCA:3 eCCA:0	1–3	34	82%†	n.r.	none
Kopek (186)	R	iCCA:26 eCCA:1	3	45	85%	10.6	6 ulcerations 3 stenosis
Momm (194)	R	iCCA:0 eCCA:13	10–12	32–56	78%	33.5	1 Grade 3 5 cholangitis
Polistina (185)	R	iCCA:0 eCCA:10	3	30*	80%**	35.5	1 ulceration 2 stenosis
Dewas (195)	R	iCCA:6 eCCA:0	3	56	100%	n.r.	n.r.
Barney (189)	R	iCCA:6 eCCA:4	3–5	45–60	100%	15.5	1 Grade 3 biliary stenosis, 1 Grade 5 liver failure
Ibarra (183)	R	iCCA:11 eCCA:0	3	22–50	55.5%	11	3 Grad 3
Liu (196)	R	iCCA: 6	3–5	50–60	93%†	n.r.	1 RILD
Jung (182)	R	iCCA:33 eCCA:25±	1–5	15–60	85%	10	6 Grade 3 (ulceration, cholangitis, stenosis, perforation)
Mahadevan (181)	R	iCCA:31 eCCA:11	3–5	24–45	88%	17	4 Grade 3 (ulceration, cholangitis, abscess)
Weiner (197)	P	iCCA:12 eCCA:0	5	40–55	91%§	13.2	1 hepatic failure§ 1 biliary stricture
Sandler (180)	R	iCCA:6 eCCA:25	5	40	78%	15.7	5 Grade ≥ 3
Gkika (176)	R	iCCA:17 eCCA: 26	3–12	21–66	78%	14	3 Grade ≥ 3
Shen (198)	R	iCCA:28	3–5	36–45	89.3%***	15	No grade 3
Brunner (177)	R	iCCA:45 eCCA:34 n.a.:7	3–17	67.2 ****	95%	15	No grade 3

R, retrospective; P, prospective; IHCCC, intrahepatic cholangiocarcinoma; EHCCC, extrahepatic cholangiocarcinoma.

*concurrent Gemcitabine.

**local response ratio.

***disease control rate.

****biological effective radiation dose.

^±^5 patients treated with conventional fractionation with a stereotactic boost.

^§^In this study SBRT was performed also in patients with hepatocellular carcinoma. LC and toxicities are reported for the whole group of patients including hepatocellular and cholangiocarcinoma.

^†^mixed collective, including hepatocellular carcinomas and metastases.

#### Brachytherapy

Several retrospective studies have evaluated the role EBRT, typically 30–40 Gy in 1.8–2 Gy with a brachytherapy boost. For BT, the dose commonly used is 15–20 Gy prescribed to the BT related PTV, generally over 2–3 treatments (HDR-BT) (130, 199). In a prospective phase I study by Mattiucci (200), investigating three different dose levels (15, 20, and 25 Gy) for HDR BT with 192Ir, recommended a dose of 25 Gy in five fractions (maximum dose level), as no dose limiting toxicities were reported up to this dose level. The median OS for these patients was 12 months. In a propensity score, matched pair analysis comparing patients receiving EBRT vs. EBRT and BT in unresectable BTC the addition of BT to EBRT had no impact on OS or disease specific survival but was associated with a better LC after 2 years (201, 202). Furthermore, BT can be used in the treatment of malignant obstructive jaundice (203). Intraluminal brachytherapy might increase the risk of cholangitis, pain, duodenopathy, and bleeding (130). Late complications such as bile duct stenosis or stricture were also observed (130, 199).

#### Particle Therapy Including Proton Therapy

Another treatment option for dose escalation in unresectable cholangiocarcinoma is the use of proton beam radiation (PBT) therapy. Initial studies (204–206) including also patients treated in palliative intent showed promising results (**Table 7**). In more recent studies, Hung et al. (209) treated 30 patients with a median radiation dose of 72.6 cobalt gray equivalents. The 1 year local control achieved was 88% similar to the SBRT series with a median OS survival of 19.3 months. Three and two patients had grade III-IV toxicities and radiation-induced liver disease. There were no deaths caused by PBT or concurrent chemotherapy. Patients who received concurrent chemotherapy had a better median PFS (12.1 vs. 4.7 months). Furthermore, in a multi-institutional phase II study patients with iCCA treated with high dose hypo-fractionated PBT achieved a LC of 94.1% at 2 years and a 2 years OS of 46.5%, with limited toxicities (4.8% grade 3 toxicity) (209). There were no grade-4 or grade-5 radiation-related toxicities (209). The Japan Carbon Ion Radiation Oncology Study Group (J-CROS) investigated the role of Carbon -ion therapy for 56 patients with intrahepatic (27 patients) and perihilar (29 patients) cholangiocarcinoma (208). Most patients were treated to a total dose of 76 GyE in 20 fractions, with a median survival of 23.8 months for intrahepatic cholangiocarcinoma and 12.6 months in perihilar disease. No patients underwent resection. There was one case of death due to liver injury and one grade 3 bile duct stenosis. Results are summarized in **Table 7**.

**Table 7:** Proton therapy in the treatment of cholangiocarcinoma.

Authors	Study	Nr of patients	Dose	Chemotherapy	OS	LC
Makita (204)	R	28	68.2 Gy RBE	15 patients	49%@1 year	67.7%@1 year
Ohkawa (205)	R	20	72.6 GyE	4 patients	Median 27.5 months (curative group) 9.6 mnths (palliative group)	60%@2 years (curative group)
Hong (207)	Ph II	37	58 GyE	none	46.5%@2years	94.1@2years
Kasuya (208)	R	56	76 Gy RBE	none	Median: 14.8 months	79.4%@1year 58.2%@2years
Shimizu (206)	R	37	72.6 GyE	16 patients	60.3%@1year	71.5%@2years (curative group)
Hung (209)	R	30	72.6 GyE	77%	19.3 months (median)	88%@1 year

GyE, Gray equivalents; RBE, relative biological effectiveness.

## Discussion and Future Perspectives

High loco-regional disease recurrence rates after R1 resection provide a rationale for using adjuvant radiotherapy with chemotherapy. Evidence from the Phase II SWOG S0809 (85)trial have demonstrated efficacy of gemcitabine and capecitabine followed by concurrent capecitabine and radiotherapy. The 2-year OS of 65% (67% and 60% in R0 and R1, respectively) and LR rates at 2 years of 11% (95% CI, 4 to18%) overall, 9% (95% CI, 2 to 17%) for R0, and 16% (95% CI, 2 to 30%) for R1 were significantly higher than the rates expected based on historical controls (84) with low toxicity rates. Currently there are no published randomized data testing the efficacy of adjuvant chemoradiation after R1 resection, as these trials are ongoing. In the phase III ACTICCA-1 trial adjuvant chemotherapy with gemcitabine and cisplatin compared to standard of care after curative intent resection of cholangiocarcinoma and muscle invasive gallbladder carcinoma has recently embedded a radiotherapy sub-study (NCT02170090 randomizing between adjuvant CRT vs. chemotherapy in EHCC and GBC (NCT02798510).

In the locally advanced inoperable cholangiocarcinoma neoadjuvant chemoradiation might confer a benefit in terms of downsizing with consecutive assessment of resectability, but this concept should be further evaluated within clinical trials. A prospective registry study is evaluating induction gemcitabine followed by 5-FU-based CCRT and maintenance capecitabine prior to LT in unresectable CCA (NCT00301379). Another randomized prospective multi -centre study is ongoing with an aim to compare 5-year OS and 3-year RFS between resection vs. CCRT followed by LT in hCCA (NCT02232932), while in a further trial the role of neoadjuvant chemoradiotherapy with concomitant oral capecitabine followed by gemcitabine in the treatment of unresectable hCCA is being evaluated prior to LT (NCT04378023).

In patients with inoperable disease, several studies have shown that dose escalation might lead to a survival benefit (175, 177). A Phase III trial from India is ongoing comparing intensity-modulated radiation therapy with weekly gemcitabine and systemic chemotherapy vs. systemic chemotherapy alone in unresectable cholangiocarcinoma (NCT02773485). Forms of safe dose escalation might include the use of a simultaneous integrated boost (SIB) or the use of brachytherapy after external radiotherapy or proton therapy with encouraging results. The use of hypo-fractionation or SBRT leads to high rates of disease control with reduced toxicity and is currently being prospectively evaluated in several trials such as the STRONG trial (NCT03307538) or the LAPIS trial (DRKS00011266). Due to the short treatment time, hypo-fractionation including SBRT can be easily incorporated into systemic treatments. Moreover, ionizing radiation, beside cytotoxicity, has been shown to additionally induce immune-modulatory effects, which trigger anti-tumor immune responses (210–215). The potentiation of anti-tumor immune responses can cause immunogenic cell death of cancer cells, change the tumor immune microenvironment, and alter antigen presentation of the tumor cells, thus enhancing immunogenicity of the tumor (216, 217). SBRT, by applying a high single dose with a few but more than one fractions, seems to have the potential to lead to an activation of specific T-cell response in the tumor (218–220). Thus, SBRT might be particularly attractive for combinations with checkpoint inhibitors. Furthermore, the short treatment interval seems to be favourable for a T-cell response. The immunomodulatory effects of SBRT are currently evaluated of LAPIS trial (DRKS00011266). In a Phase I/II Study (NCT04068194) patients with advanced/metastatic solid tumors and hepatobiliary malignancies including cholangiocarcinomas are treated with hypo-fractionated radiation in combination with M3814 and avelumab another trial is investigating the combination of hypo-fractionated RT with modified Immune Cells (Autologous Dendritic Cells) and a Vaccine (Prevnar) in patients with liver tumos (NCT03942328) including CCAs. The combination of chemotherapy with normo-fractionated RT or SBRT and the anti-PD-1 Antibody Camrelizumab is currently investigated in another prospective trial (NCT03898895). However, the mode of cell death and the systemic effects induced by ionizing irradiation are not uniform, and it clearly depends on the irradiation dose, the fractionation regimen, and the genetic repertoire of the irradiated cells (221).

In the past decade, the genetic landscape of cholangiocarcinoma subtypes has evolved and promising molecular targets for precision medicine have been identified. As the molecular classification and liquid biopsies are being gradually integrated in the treatment of solid tumors, efforts should be focused in identifying biomarkers to aid patients’ selection for radiotherapy or combined treatments such as SBRT with checkpoint inhibitors.

## Author Contributions

All authors contributed to the article and approved the submitted version.

## Funding

MH is supported by funding from the NIHR Biomedical Research Centre at University College London Hospitals NHS Foundation Trust.

## Conflict of Interest

The authors declare that the research was conducted in the absence of any commercial or financial relationships that could be construed as a potential conflict of interest.
